# Polycomb Repressive Complex 2-mediated histone modification H3K27me3 is associated with embryogenic potential in Norway spruce

**DOI:** 10.1093/jxb/eraa365

**Published:** 2020-09-07

**Authors:** Miyuki Nakamura, Rita A Batista, Claudia Köhler, Lars Hennig

**Affiliations:** 1 Department of Plant Biology, Uppsala BioCenter, Swedish University of Agricultural Sciences and Linnean Center for Plant Biology, Sweden; 2 Trinity College Dublin, Ireland

**Keywords:** Embryogenic callus, gymnosperm, H3K27me3, histone modification, Norway spruce, somatic embryos

## Abstract

Epigenetic reprogramming during germ cell formation is essential to gain pluripotency and thus embryogenic potential. The histone modification H3K27me3, which is catalysed by the Polycomb repressive complex 2 (PRC2), regulates important developmental processes in both plants and animals, and defects in PRC2 components cause pleiotropic developmental abnormalities. Nevertheless, the role of H3K27me3 in determining embryogenic potential in gymnosperms is still elusive. To address this, we generated H3K27me3 profiles of Norway spruce (*Picea abies*) embryonic callus and non-embryogenic callus using CUT&RUN, which is a powerful method for chromatin profiling. Here, we show that H3K27me3 mainly accumulated in genic regions in the Norway spruce genome, similarly to what is observed in other plant species. Interestingly, H3K27me3 levels in embryonic callus were much lower than those in the other examined tissues, but markedly increased upon embryo induction. These results show that H3K27me3 levels are associated with the embryogenic potential of a given tissue, and that the early phase of somatic embryogenesis is accompanied by changes in H3K27me3 levels. Thus, our study provides novel insights into the role of this epigenetic mark in spruce embryogenesis and reinforces the importance of PRC2 as a key regulator of cell fate determination across different plant species.

## Teaser

PRC2-mediated H3K27me3 is associated with embryogenic potential in Norway spruce, and the early phase of somatic embryogenesis is accompanied by changes in H3K27me3 levels.

## Introduction

**Fig. 1.**
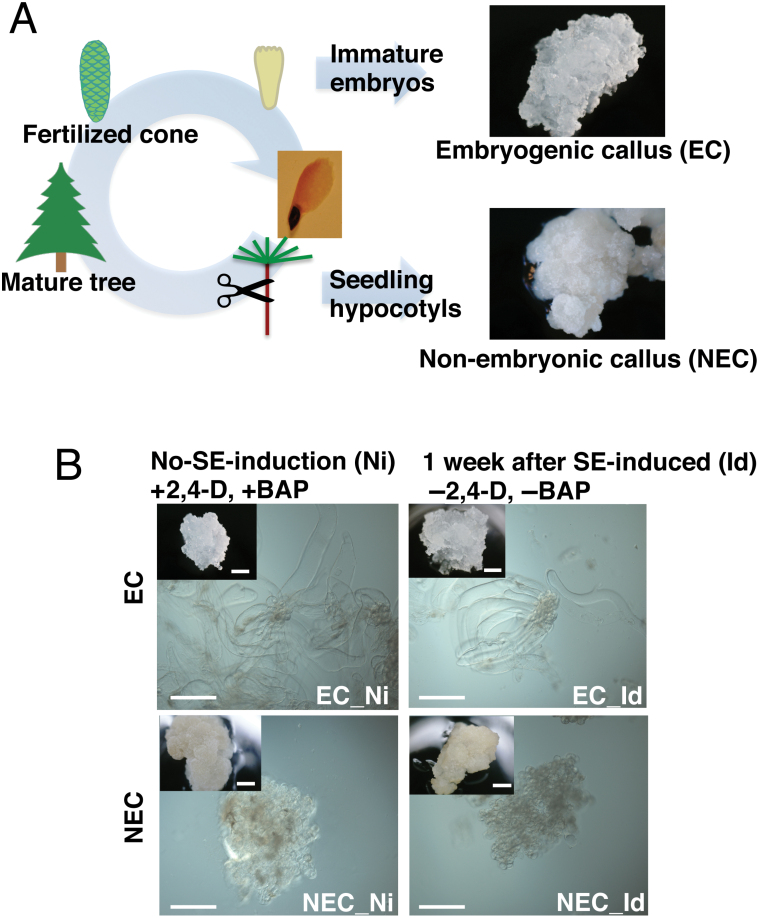
Embryogenic callus (EC) and Non-embryogenic callus (NEC) in Norway spruce. (A) Explants from immature embryo form EC and explants from seedling hypocotyl form NEC. (B) Morphology of EC and NEC before and 1 week after somatic embryogenesis induction. Both types of calli can be induced and maintained in the presence of 9 µM 2,4-dichlorophenoxyacetic acid (2,4-D) and 4.4 µM 6-benzylaminopurine (BAP). Transferring to medium without 2,4-D or BAP triggers somatic embryogenesis in EC. Scale bar: 200 μm; inset scale bar: 2 mm.

Micropropagation through somatic embryogenesis in spruce species was established nearly three decades ago, and nowadays this method is commercially applied to propagate several coniferous species (reviewed in Klimaszewska *et al.*, 2015). This technology enables an efficient proliferation of spruce individuals, bypassing the long reproductive cycle of this species. Norway spruce (*Picea abies*) is a common conifer species that is ecologically and economically important in Europe. In Norway spruce, two different types of callus can be generated: embryogenic callus (EC), usually induced from immature embryos, and non-embryogenic callus (NEC), induced from vegetative seedling tissues (Nagmani *et al.*, 1987; Becwar *et al*., 1988; Verhagen & Wann, 1989). Interestingly, the success rate of EC formation is largely dependent on the tissue of the original embryos and their developmental stages (Becwar *et al*., 1988; Ramarosandratana & Van Staden, 2003). Immature embryos have a higher potential for EC formation compared with mature tissues (Jain *et al.*, 1988; von Arnold *et al.*, 1995). After callus formation, EC and NEC are different in their appearance and biochemical properties (Becwar *et al*., 1988) (Fig. 1A), but both can be maintained in a growth medium containing 2,4-dichlorophenoxyacetic acid (2,4-D) and 6-benzylaminopurine (BAP). Transferring these calli to a medium without these phytohormones induces somatic embryogenesis exclusively in EC, showing that EC and NEC have distinct embryogenic potentials (Nagmani *et al.*, 1987; Becwar *et al*., 1988). Nevertheless, little is known about the molecular mechanisms underlying the distinct embryonic competencies between EC and NEC.

Polycomb repressive complex 2 (PRC2) contains histone methyltransferase activity, and is responsible for the tri-methylation of lysine 27 of histone H3 (H3K27me3). This histone modification is a repressive epigenetic mark observed in developmental regulatory genes and environmental responsive genes (reviewed in (Schwartz & Pirrotta, 2007; Hennig & Derkacheva, 2009; Margueron & Reinberg, 2011). The H3K27me3 mark plays a crucial role in cell differentiation and tissue morphogenesis. In Arabidopsis, H3K27me3 profiles change when cells convert from a differentiated to a multipotency state, and vice versa (Lafos *et al.*, 2011; He *et al.*, 2012). Depletion of PRC2 function causes disruption of post-embryonic development, resulting in the formation of callus- or embryo-like cell masses after germination (Chanvivattana *et al.*, 2004; Bouyer *et al.*, 2011). Strikingly, in PRC2 mutants, leaf tissue explants have no capacity of callus formation, while callus formation from root is unaffected (He *et al.*, 2012). Because the process of callus formation from leaves transits through a root-like developmental program (Sugimoto *et al.*, 2010), callus formation has been recently considered as a transdifferentiation process rather than a dedifferentiation process (Sugimoto *et al.*, 2011). Thus, the callus formation process is different depending on the original tissue. Interestingly, PRC2 mutants show a higher competency of somatic embryo induction from vegetative tissues when compared with wild-type plants (Mozgová *et al.*, 2017). This suggests that H3K27me3 plays a crucial role in repression of pluripotency in Arabidopsis. In contrast, to date, knowledge about the influence of chromatin status, in particular of H3K27me3 deposition, in gymnosperm embryogenesis is very limited.

Here, we surveyed the differences of H3K27me3 deposition in EC and NEC of Norway spruce. Most genome-wide analyses of histone modifications in plants have been accomplished in species with relatively small genomes, with the exception of maize, barley, and wheat (Zhang *et al.*, 2007; Makarevitch *et al.*, 2013; Widiez *et al.*, 2014; Baker *et al.*, 2015; Hussey *et al.*, 2017; Latrasse *et al.*, 2017; Chica *et al.*, 2017; Lü *et al.*, 2018; Qi *et al.*, 2018; Li *et al.*, 2019; Montgomery *et al.*, 2020). In contrast to many angiosperm studies, genome-wide chromatin profiling in other plant species has been limited to moss, liverwort, and unicellular algae (Widiez *et al.*, 2014; Mikulski *et al.*, 2017; Montgomery *et al.*, 2020). Gymnosperm species have generally very large (4–30 Gbp) and poorly assembled genomes, making this type of profiling laborious and challenging. To explore the roles of H3K27me3 in embryogenic competency of gymnosperms and chromatin dynamics during early phases of somatic embryogenesis, we identified genes with H3K27me3 marks in EC and NEC using CUT&RUN, which is an antibody-based technology for profiling targeted DNA–protein interactions (Skene & Henikoff, 2017). This method reduces background signals compared with the conventional chromatin immunoprecipitation (ChIP) method (Skene *et al.*, 2017) and is thus more suitable for whole genome analyses of histone modifications in organisms with large genome, such as gymnosperms.

In both EC and NEC, the H3K27me3 mark was enriched in genic regions, rather than in intergenic regions or transposable elements. Nearly half of H3K27me3-enriched genes were common between EC and NEC; however, many genes showed lower H3K27me3 levels in EC before somatic embryo induction when compared with other samples. For example, some homologues of *AINTEGUMENTA-LIKE* (*AIL*) and *ABSCISIC ACID INSENSITIVE 3* (*ABI3*) transcription factor genes display low H3K27me3 in EC compared with NEC. Interestingly, a 1-week period in embryo-inducing medium was sufficient to provoke alterations in H3K27me3 profiles of both EC and NEC. Notwithstanding, these two types of callus displayed large differences in H3K27me3 profiles in response to somatic embryo induction. Thus, we revealed that two different types of calli in *Picea abies* have distinct chromatin profiles, suggesting that the genome-wide H3K27me3 status in EC underlies its high pluripotency of cell differentiation.

## Materials and methods

### Plant materials and growth conditions

The Norway spruce (*Picea abies* L. Karst) embryogenic culture cell line (EC) was established from immature embryos by a previously described method (von Arnold & Clapham, 2008). EC was established and proliferated on half-strength LP medium at 22 °C in the dark. For somatic embryo induction, calli were transferred into solidified BMI-SI medium (von Arnold & Clapham, 2008) and were harvested 1 week later. Two-month-old seedlings were grown in quarter-strength SH medium (von Arnold & Clapham, 2008). For NEC formation, explants from hypocotyls of 2-month-old seedlings were cultured on the half-strength LP medium at 22 °C in the dark. NEC were generated in April 2017 and harvested in February 2018.

### Imaging of culture cell morphology

Subcultured calli from EC and NEC were crushed with forceps in 0.3 M sorbitol solution. Images were captured through a differential interference contrast microscope (Axioscope A1, Zeiss, Germany) and a Leica camera DFC490.

### CUT&RUN and sequencing

Nuclei were isolated as described previously with the exception that the MEB buffer contained no EGTA (Shu *et al.*, 2014). CUT&RUN (Skene & Henikoff, 2017) was carried out using isolated nuclei (from EC and NEC before and after induction and from seedlings) as described previously with modifications as follows. Triton X-100 was added to the washing buffer at a final concentration of 0.1%. After the extraction of digested DNA, larger fragments were removed from extracted DNA by ×0.6 volume Agencourt AMPure XP (Beckman Coulter). Subsequently, the DNA was recovered by ×3.0 volume AMpure XP. Major modifications from the original CUT&RUN protocol are as follows. Extracted nuclei were immobilized onto concanavalin A (ConA) beads. Chromatin binding to beads was confirmed by comparing recovered amounts of genomic DNA between flow-through and pull-down fractions. To avoid chelation of calcium ions, which are required for ConA bead binding, MEB buffer without EGTA was used for nuclear extraction. Triton X-100 was added to the washing buffer to ensure permeability of pA-MNase. After pA-MNase digestion, DNA size distribution corresponding to mono-, di-, and tri-nucleosomes was confirmed using the Bioanalyzer system (Agilent Technologies). Anti-H3K27me3 antibody was used (Cell Signaling Technology cat. no. 9733S). The libraries were prepared using MicroPlex Library Preparation Kit v2 (Diagenode) following the manufacturer’s instructions. Paired-end reads of 125 bp were sequenced with an Illumina HiSeq2500 on the National Genomics Infrastructure—SciLifeLab. The same amount of spike-in DNA (*Saccharomyces cerevisiae* genomic DNA) was added to each sample after extracting MNase-digested DNA. The read counts of spike-in DNA, which mapped to the *S. cerevisiae* reference genome, were well correlated with total read counts in each sample.

### Data analysis

H3K27me3 signal peaks were called as follows. (i) The paired-end reads were separately mapped by BWA (v0.7.17) (Li & Durbin, 2009) to the *P. abies* whole-genome v1.0 (Congenie.org) and merged if they were mapped in the same contigs. (ii) The merged fragments with a size longer than 700 bp were discarded. (iii) The contigs of genome sequences were split by 500 bp bins. (iv) The read numbers overlapping each bin were counted and *P*-values were calculated using a binominal test by each bin. (v) Obtained *P*-values were adjusted by p.adjust packages in R using the Benjamini–Hochberg method. (vi) The bins with false discovery rate ≤10^−25^ were selected. Peaks were merged if their distances were shorted than 100 bp. The genes that overlapped with H3K27me3 peaks were defined as H3K27me3-enriched genes. The H3K27me3 signal intensity per gene was defined as reads per kilobase per billion mapped reads over gene regions, including 500 bp extensions in both upstream and downstream orientations. For genomic features, transposable element sequences were annotated as described before (Nakamura *et al.*, 2019). For drawing signal distribution over genes, deepTools computeMatrix and plotHeatmap were used (Ramírez *et al.*, 2014). Shannon entropy was calculated by the R package ‘entropy’ using transcriptome data retrieved from the ConGenIE resource (http://congenie.org/). To calculate the Shannon entropy the following transcriptome datasets were used for organ specificity: ENA ID: ERS235801 (male flower), ERS235809 (female flower), ERS235803 (needles from 2009), ERS235807 (pineapple galls), ERS235808 (buds, early season), ERS235811 (stem from vegetative shoots), ERS235802 (vegetative shoots), and ERS235821 (wood, June), and for condition specificity: ENA ID: ERS235803 (needles from 2009), ERS235804 (needles from 2008), ERS235805 (infected needles), ERS235814 (needles from dried twig), ERS235815 (needles girdled twig), ERS235817 (early morning needles), ERS235818 (mid-day needles), ERS235819 (late afternoon needles), and ERS235820 (night needles) (Nystedt *et al.*, 2013). Homologues of Arabidopsis proteins were identified as previously described (Nakamura *et al.*, 2019).

### Phylogenetic analysis of H3K27 methyltransferase homologues

CLF, SWN, and MEA amino acid sequences were obtained from TAIR10 and used as query sequences to identify E(z) homologues in the plant kingdom, using tblastx (Camacho *et al.*, 2009). The dataset of coding sequences (CDS) in PLAZA gymnosperm (v1.0), Plaza Monocots (v4.5), ConGenIE (v1.0), Coge (https://genomevolution.org/coge/), Phytozome (v1), and NCBI, were searched for homologues in *Amborella trichopoda*, *Persea americana*, *Populus trichocarpa*, *Oryza sativa* ssp. *japonica*, *Zea mays*, *Picea abies*, *Picea glauca*, *Picea sitchensis*, *Pinus pinaster*, *Pinus sylvestris*, *Pseudotsuga menziesii*, *Taxus baccata*, *Cycas micholitzii*, *Ginkgo biloba*, *Gnetum montanum*, *Selaginella moellendorffii*, *Sphagnum fallax*, *Marchantia polymorpha*, *Physcomitrella patens*, and *Chlamydomonas reinhardtii* (Goodstein *et al.*, 2012; Van Bel *et al.*, 2018). Amino acid sequences of the SET domain were aligned using ClustalW and the phylogenetic tree was constructed by PHYML and drawn in MEGA7.0 (Thompson *et al.*, 1994; Guindon *et al.*, 2010; Kumar *et al.*, 2016). The E(z) of *Drosophila melanogaster* and MA_9310110g0010, which is a homologue of Arabidopsis SDG7, were used as an outgroup.

### Gene ontology analysis

A list of Arabidopsis genes homologous to the *P. abies* genes of interest was used for gene ontology (GO) analysis. The Arabidopsis GO annotation was downloaded from TAIR (https://www.arabidopsis.org). The hypergeometric *P*-values were calculated based on the frequency of each GO term in *P. abies* genes of interest with Arabidopsis homologues over the whole set of *P. abies* genes with Arabidopsis homologues. Enriched GO terms were summarized using REVIGO (Supek *et al.*, 2011).

### DNA motif identification

After mapping, the read counts per base pair were normalized by total reads and then converted into Bedgraph format by Bedtools (Quinlan & Hall, 2010). Subsequently, the regions with more than 8.0×10^−7^ read/total reads were considered signal peaks. If the distance between adjacent peaks was less than 101 bp, the peaks were merged. To calculate the motif enrichment, background DNA sequences were randomly picked from the *P. abies* genome to have the same length distribution as the peak regions. Selected DNA sequences were used as a query to run MEME-ChIP (Machanick & Bailey, 2011). The JASPAR core (2018) plant dataset was used as known motifs.

### Mapping data

Mapping data of each tissue are listed in Supplementary Table S1 at *JXB* online.

### Reverse transcription–quantitative PCR

Total RNA was extracted as previously described (Nakamura *et al.*, 2019). The cDNA was synthesized by RevertAid First Strand cDNA Synthesis Kit (Thermo Fisher Scientific) with both oligo(dT) and oligo hexamer primers. Quantitative real-time PCR was performed as described (Nakamura *et al.*, 2019). Primers used are listed in Supplementary Table S2.

## Results

### Characterization of H3K27me3-rich regions in Norway spruce

**Fig. 2.**
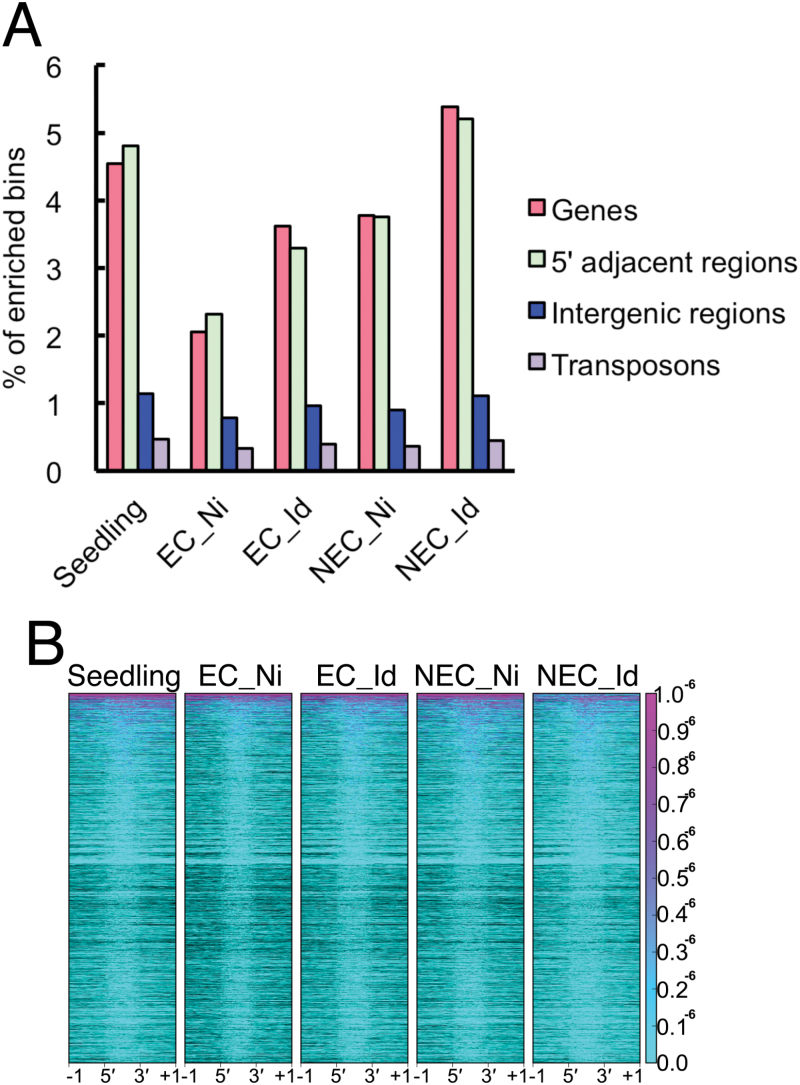
H3K27me3 accumulates in genic regions. (A) H3K27me3-enriched genomic features. (B) H3K27me3 accumulation over target genes ±1 kb adjacent regions.

To profile the H3K27me3 histone modification in the Norway spruce genome (19.6 Gbp), we adapted the previously developed CUT&RUN method (Skene & Henikoff, 2017) to this species. Given our interest in unravelling the role of this histone modification on the embryogenic potential of Norway spruce, we sampled EC and NEC, before and after somatic embryo induction (Fig. 1B). Our main focus was on early changes, before cell morphological changes were visible. We therefore sampled calli 1 week after somatic embryo induction prior to morphological changes (Filonova *et al.*, 2000). EC consisted of two different cell types: elongated cells and small and compacted cells (Fig. 1B) (Filonova *et al.*, 2000). On the other hand, NEC consisted of only round small cells. After 2 weeks of SE induction, compacted cells of EC proliferated and aggregated to generate proembryonic cell masses (Filonova *et al.*, 2000), whereas cells of NEC turned brown (see Supplementary Fig. S1). As a control, we also sampled seedlings. Then, we investigated the genomic features that overlapped with H3K27me3 peaks. We found that H3K27me3 was highly enriched in coding regions and presumable promoter regions, across all sample types (Fig. 2A–B). On the other hand, repetitive sequences and intergenic regions were under-represented among H3K27me3-enriched regions (Fig. 2A). These observations are consistent with previous studies in angiosperms, moss, and unicellular algae (Zhang *et al.*, 2007; Makarevitch *et al.*, 2013; Widiez *et al.*, 2014; Baker *et al.*, 2015; Hussey *et al.*, 2017; Latrasse *et al.*, 2017; Mikulski *et al.*, 2017; Chica *et al.*, 2017; Lü *et al.*, 2018; Qi *et al.*, 2018; Li *et al.*, 2019). This shows that (i) the CUT&RUN method can be successfully applied to large genomes, such as that of Norway spruce, and (ii) despite the significant differences in genome size between angiosperms and gymnosperms, deposition of H3K27me3 is detected in similar genomic features across different plant species.

**Fig. 3.**
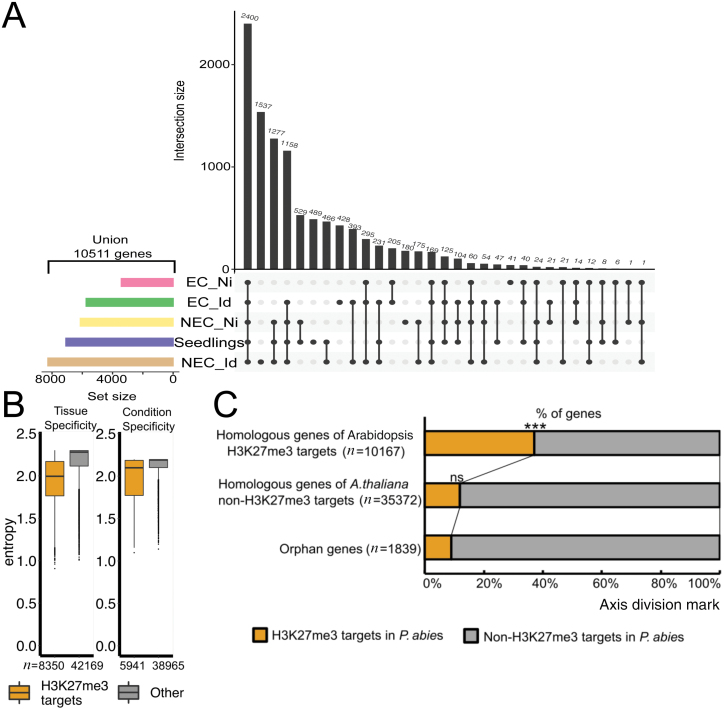
Characterization of H3K27me3 target genes regarding their expression specificity and conservation. (A) UpSet plot of H3K27me3-enriched genes of five different tissues. (B) Shannon entropy distribution between subsets of H3K27me3-target and non-target genes. (C) Conservation of H3K27me3 target genes between Arabidopsis and Norway spruce. Statistical significance was determined using the hypergeometric test: ***P<0.0005; ns: *P*>0.05.

Among the five tissue samples, a total of 10 511 genes had H3K27me3 peaks within the gene body or in adjacent regions (Fig. 3A). Each tissue had more than 3000–8000 genes marked with H3K27me3 (Fig. 3A). NEC tissue subject to somatic embryo induction (NEC_Id) had the highest number of H3K27me3-marked genes. On the other hand, non-induced EC (EC_Ni) had only 3422 H3K27me3-marked genes. Among all five samples there were 2400 genes that overlapped. The H3K27me3 modification is frequently present in developmental regulatory genes, in both animals and plants (Hennig & Derkacheva, 2009; Margueron & Reinberg, 2011). To test the developmental roles of H3K27me3 in Norway spruce, we investigated the expression specificity of H3K27me3-enriched genes across different tissues or environmental conditions. We calculated the Shannon entropy between H3K27me3-enriched genes and non-enriched genes. This measure can be used to estimate the specificity of expression of a given gene (Schug *et al.*, 2005). We used eight different tissue/organ transcriptome datasets (male cone, female cone, wood, stems, among others), and nine transcriptome datasets of different conditions of needles (daytime, infection, wounding, among others) (see ‘Materials and methods’ for details). In both cases, the subset of H3K27me3-enriched genes showed a lower Shannon entropy compared with the subset that was not marked by H3K27me3. These results indicate that genes targeted by H3K27me3 are expressed in specific organs or conditions. Thus, H3K27me3-enriched genes in gymnosperms show responsiveness to developmental and environmental regulation, similarly to what is observed in angiosperms (Zhang *et al.*, 2007; Makarevitch *et al.*, 2013; Liu *et al.*, 2015; Akter *et al.*, 2019).

Next, we wondered whether the sets of genes marked with H3K27me3 are similar between angiosperms and gymnosperms. To test this, we assigned an Arabidopsis homologue to each *P. abies* gene. Approximately 65% of the total *P. abies* gene models had homology to at least one Arabidopsis gene based on a BLAST search. We then grouped the Arabidopsis homologues based on the presence of H3K27me3. Nearly 40% of *P. abies* genes showing homology to an Arabidopsis H3K27me3-marked gene were also H3K27me3-marked (Fig. 3C). This ratio is significantly higher when compared with that in *P. abies* homologous genes of non-H3K27me3 targets in Arabidopsis (less than 15%). Approximately 10% of orphan genes, which are specific for the gymnosperm lineage, had H3K27me3 marks (Fig. 3C), suggesting that newly evolved genes become integrated into the transcriptional network regulated by PRC2.

Proliferation of EC and NEC requires phytohormones, such as 2,4-D and BAP. A previous study in Arabidopsis showed that gene families related to auxin pathways, such as *YUCCAs*, *IAAs*, and *PIN-FORMEDs*, are targeted by H3K27me3, but *ARFs* are not (Lafos *et al* 2011). To investigate whether homologues of these gene families in Norway spruce have a similar tendency, we scored the percentage of genes with H3K27me3 in auxin-related gene families. Interestingly, we found that Norway spruce *ARF* homologues also lacked H3K27me3, while *YUCCA* homologues tended to be marked by H3K27me3, similarly to Arabidopsis. In contrast, *PIN-FORMED* homologues were depleted of H3K27me3 in all tissues examined (see Supplementary Fig. S2).

PRC2 core components are conserved throughout the plant kingdom (Hennig & Derkacheva, 2009). Angiosperm genomes have three to four H3K27 methyltransferase genes, which are called E(z) homologues (Hennig & Derkacheva, 2009). In Arabidopsis, the E(z) homologues are *SWINGER* (*SWN*), *CURLY LEAF* (*CLF*), and *MEDEA* (*MEA*). The latter is expressed only during the reproductive phase and is responsible for parent-of-origin-specific gene expression in the endosperm (reviewed in Batista & Köhler, 2020). Because previous phylogenetic analyses of E(z) homologues lack information on gymnosperm species, we performed a phylogenetic analysis of H3K27 methyltransferase genes of gymnosperms. All the species examined here had at least two putative H3K27 methyltransferase genes (see Supplementary Fig. S3), which are in the same clade as the Arabidopsis *CLF* gene. In Arabidopsis, CLF and SWN have partially redundant functions (Chanvivattana *et al.*, 2004). Interestingly, we could only detect basal angiosperm, eudicot, and monocot homologues within the SWN orthologue clade (Supplementary Fig. S3). Thus, these data suggest that E(z) homologues underwent radiation and subfunctionalization in angiosperms after the divergence of angiosperms and gymnosperms.

In Arabidopsis, some H3K27me3 targets are known to be associated with Polycomb response elements (PRE), *cis*-regulatory elements responsible for the targeting of PRC2 (Xiao *et al.*, 2017; Zhou *et al.*, 2018). To test whether specific DNA sequences are associated with H3K27me3 deposition in gymnosperms, we performed a *de novo* motif identification using the MEME-ChIP algorithm (Machanick & Bailey, 2011). In both EC and NEC, most identified motifs were short AT-rich features, including a sequence similar to the target site of the DOF-domain-binding transcription factor (AT5G66940) (see Supplementary Fig. S4). Interestingly, we could also identify a motif partially similar to the one used by the Arabidopsis histone demethylase REF6 to bind its targets. Other DNA motifs were not shared between EC and NEC. Collectively, these data indicate that H3K27me3 in *P. abies* tends to localize at AT-rich sequences; nevertheless, additional studies will be required to further characterize and validate *cis-*regulatory elements responsible for PRC2 targeting and subsequent H3K27me3 deposition.

### H3K27me3 profiles and response to somatic embryo induction in EC are largely different from those in NEC

**Fig. 4.**
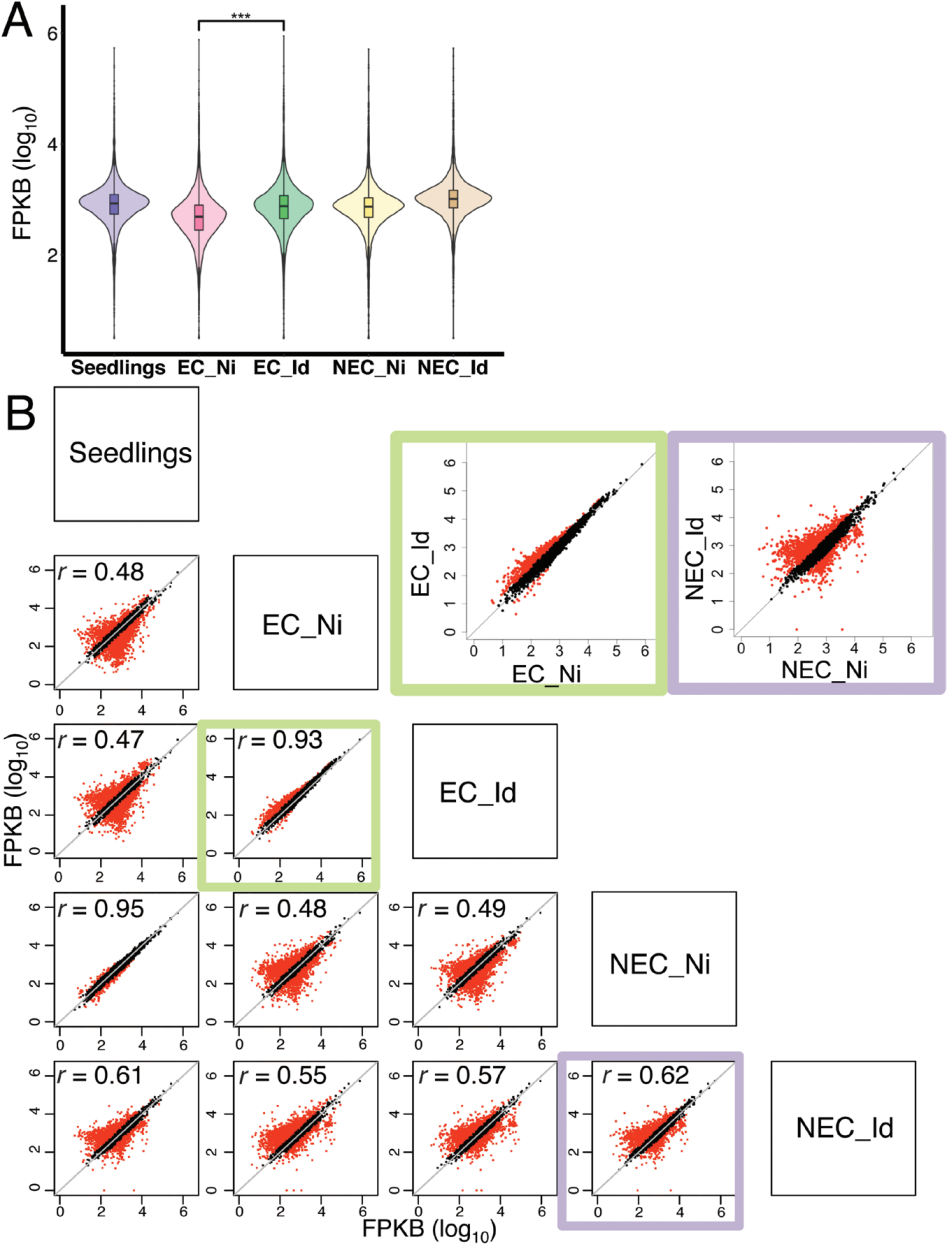
Non-induced EC shows reduced H3K27me3 signals. (A) Distributions of H3K27me3 signal intensity. ***P<0.0005, statistical significance using a paired Student’s *t*-test. (B) Pair-wise comparisons of H3K27me3 signals in target genes. Red dots indicate genes with a difference in H3K27me3 accumulation of twice or more.

Pervious work revealed that H3K27me3 is present in callus tissue of Arabidopsis, and there is even *de novo* H3K27me3 present in callus (He *et al.*, 2012). Consistent with this, both spruce EC and spruce NEC had detectable H3K27me3 (Figs 1–3). Nevertheless, the non-induced EC sample (EC_Ni) had the lowest levels of H3K27me3, which increased after embryogenesis induction (Fig. 4A). To directly compare the H3K27me3 signal per gene, and between different tissues, we plotted H3K27me3 reads in fragments per kilobase billion (FPKB) of each H3K27me-enriched gene in a pair-wise manner (Fig. 4B). We identified a considerable subset of genes showing increased H3K27me3 levels upon induction in EC, while only a few genes showed reduced H3K27me3 after induction (Fig. 4B, EC_Id versus EC_Ni). Unexpectedly, H3K27me3 profiles were largely similar between non-induced NEC and seedlings (Fig. 4B, seedling versus NEC_Ni), indicating that callus formation in NEC does not have a major impact on H3K27me3 accumulation. Nevertheless, somatic embryo induction of NEC also caused a dramatic alteration of H3K27me3 trends (Fig. 4B, NEC_Id versus NEC_Ni). Thus, these data indicate that the genes marked with H3K27me3 are largely different between EC and NEC. Moreover, H3K27me3 changes in response to somatic embryo induction are also distinct between EC and NEC.

To address the functional attributes of differentially marked genes (DMGs) between EC and NEC, we identified orthologues of DMGs in Arabidopsis and then analysed whether specific GO terms were enriched among these orthologous genes. Among the 789 genes that were H3K27me3-marked in non-induced EC, but not marked in non-induced NEC, GO terms related to response to metabolism, and in particular flavonoid metabolism, were most highly enriched (Table 1). On the other hand, somatic embryogenesis, positive regulation of cell differentiation, and negative regulation of cell proliferation were among the GO terms enriched in the 3498 genes that were H3K27me3 marked in non-induced NEC, but not marked in non-induced EC (Table 2). These data indicate that genes required to confer somatic embryogenesis competence are repressed by H3K27me3 in non-induced NEC tissues in Norway spruce (Table 2). After somatic embryo induction, 1741 genes more than doubled the signal of H3K27me3 in EC. In this gene subset, GO terms belonging to cell wall metabolism were enriched (Table 3).

**Table 1.** The GO enrichment of genes marked by H3K27me3 specifically in EC_Ni but not in NEC_Ni

GO term	Annotation	Frequency in sample	Enrichment	*P*-value
Biological process				
GO:0009892	Negative regulation of metabolic process	6	16.3	9.35×10^−8^
GO:0009800	Cinnamic acid biosynthetic process	6	11.4	1.13×10^−6^
GO:1901601	Strigolactone biosynthetic process	5	8.0	4.28×10^−5^
GO:0009411	Response to UV	11	6.8	1.33×10^−7^
GO:0010089	Xylem development	6	5.3	1.58×10^−4^
GO:0009813	Flavonoid biosynthetic process	17	5.1	1.55×10^−8^
GO:0042744	Hydrogen peroxide catabolic process	18	4.7	1.98×10^−8^
GO:0002239	Response to oomycetes	6	4.6	3.74×10^−4^
GO:0009739	Response to gibberellin stimulus	12	4.6	3.13×10^−6^
Molecular function				
GO:0016711	Flavonoid 3′-monooxygenase activity	11	11.7	2.94×10^−10^
GO:0042973	Glucan endo-1,3-β-D-glucosidase activity	5	8.0	4.28×10^−5^
GO:0008134	Transcription factor binding	5	5.4	3.81×10^−4^
GO:0004620	Phospholipase activity	5	4.9	6.06×10^−4^
GO:0008757	*S*-Adenosylmethionine-dependent methyltransferase activity	7	4.4	2.47×10^−4^
GO:0016747	Transferase activity, transferring acyl groups other than amino-acyl groups	9	4.2	7.15×10^−5^
GO:0020037	Heme binding	48	4.1	3.27×10^−16^
GO:0016762	Xyloglucan:xyloglucosyl transferase activity	6	4.1	7.77×10^−4^
GO:0000977	RNA polymerase II regulatory region sequence-specific DNA binding	7	3.7	7.15×10^−4^
GO:0019825	Oxygen binding	29	4.0	1.91×10^−10^
GO:0004601	Peroxidase activity	18	4.0	2.14×10^−7^
GO:0005506	Iron ion binding	30	3.6	1.39×10^−9^

**Table 2.** The GO enrichment of genes marked by H3K27me3 specifically in NEC_Ni but not in EC_Ni

GO term	Annotation	Frequency in sample	Enrichment	*P*-value
Biological process				
GO:0033231	Carbohydrate export	7	7.8	1.93×10^−6^
GO:0045597	Positive regulation of cell differentiation	8	7.8	5.07×10^−7^
GO:0035336	Long-chain fatty-acyl-CoA metabolic process	7	6.1	1.49×10^−5^
GO:0010345	Suberin biosynthetic process	36	5.4	3.11×10^−17^
GO:0009963	Positive regulation of flavonoid biosynthetic process	6	5.2	1.27×10^−4^
GO:0016102	Diterpenoid biosynthetic process	15	4.6	1.68×10^−7^
GO:0010262	Somatic embryogenesis	9	3.7	1.56×10^−4^
GO:0052325	Cell wall pectin biosynthetic process	8	3.6	3.93×10^−4^
GO:0008285	Negative regulation of cell proliferation	7	3.4	9.88×10^−4^
GO:0048544	Recognition of pollen	29	3.2	1.82×10^−8^
GO:0010268	Brassinosteroid homeostasis	16	3.0	2.37×10^−5^
Molecular function				
GO:0009674	Potassium:sodium symporter activity	5	7.1	5.16×10^−5^
GO:0103075	Indole-3-pyruvate monooxygenase activity	6	6.7	2.25×10^−5^
GO:0080019	Fatty-acyl-CoA reductase (alcohol-forming) activity	15	6.5	9.07×10^−10^
GO:0102406	Omega-hydroxypalmitate *O*-sinapoyl transferase activity	8	5.9	6.03×10^−6^
GO:0047560	3-Dehydrosphinganine reductase activity	9	4.8	1.44×10^−5^
GO:0034768	(*E*)-β-Ocimene synthase activity	25	4.8	1.63×10^−11^
GO:0009905	*ent*-Copalyl diphosphate synthase activity	15	4.7	1.27×10^−7^
GO:0051119	Sugar transmembrane transporter activity	20	4.5	3.74×10^−9^
GO:0008429	Phosphatidylethanolamine binding	10	4.3	1.80×10^−5^
GO:0008395	Steroid hydroxylase activity	10	4.3	1.80×10^−5^
GO:0047372	Acylglycerol lipase activity	15	4.3	4.86×10^−7^
GO:0016628	Oxidoreductase activity, acting on the CH-CH group of donors, NAD or NADP as acceptor	8	4.2	1.19×10^−4^
GO:0050105	L-Gulonolactone oxidase activity	14	4.0	2.52×10^−6^
GO:0047890	Flavanone 4-reductase activity	8	3.8	2.51×10^−4^
GO:0045552	Dihydrokaempferol 4-reductase activity	8	3.8	2.51×10^−4^
GO:0102483	Scopolin β-glucosidase activity	16	3.5	3.47×10^−6^
GO:0052716	Hydroquinone:oxygen oxidoreductase activity	23	3.2	3.64×10^−7^
GO:0016747	Transferase activity, transferring acyl groups other than amino-acyl groups	35	3.1	1.07×10^−9^
Cellular component				
GO:0000325	Plant-type vacuole	9	3.9	9.87×10^−5^
GO:0031012	Extracellular matrix	11	3.8	3.12×10^−5^

**Table 3.** The GO enrichment of genes that gain H3K27me3 after somatic embryo induction in EC

GO term	Annotation	Frequency in sample	Enrichment	*P*-value
Biological process				
GO:0016102	Diterpenoid biosynthetic process	12	7.8	5.23×10^−9^
GO:0002679	Respiratory burst involved in defense response	6	7.4	1.63×10^−5^
GO:0080163	Regulation of protein serine/threonine phosphatase activity	7	6.7	1.06×10^−5^
GO:0006666	3-Keto-sphinganine metabolic process	5	5.7	2.36×10^−4^
GO:0048544	Recognition of pollen	19	4.4	2.04×10^−8^
GO:0006949	Syncytium formation	10	4.4	2.15×10^−5^
GO:0009607	Response to biotic stimulus	6	4.0	8.11×10^−4^
GO:0009625	Response to insect	12	3.9	1.74×10^−5^
GO:0042744	Hydrogen peroxide catabolic process	35	3.8	1.43×10^−11^
GO:0042545	Cell wall modification	14	3.6	1.08×10^−5^
GO:0010089	Xylem development	9	3.3	5.19×10^−4^
GO:0009740	Gibberellic acid mediated signaling pathway	17	3.1	1.29×10^−5^
Molecular function				
GO:0009905	*ent*-Copalyl diphosphate synthase activity	12	8.0	4.09×10^−9^
GO:0004864	Protein phosphatase inhibitor activity	8	6.1	7.55×10^−6^
GO:0010333	Terpene synthase activity	16	6.1	1.93×10^−9^
GO:0047560	3-Dehydrosphinganine reductase activity	5	5.7	2.36×10^−4^
GO:0010427	Abscisic acid binding	7	5.4	4.81×10^−5^
GO:0050502	*cis*-Zeatin *O*-β-D-glucosyltransferase activity	6	5.1	1.78×10^−4^
GO:0052716	Hydroquinone:oxygen oxidoreductase activity	13	3.8	1.03×10^−5^
GO:0045330	Aspartyl esterase activity	14	3.8	5.56×10^−6^
GO:0030599	Pectinesterase activity	14	3.7	8.19×10^−6^
GO:0004601	Peroxidase activity	39	3.6	5.01×10^−12^
GO:0016614	Oxidoreductase activity, acting on CH-OH group of donors	10	3.4	2.31×10^−4^
GO:0030246	Carbohydrate binding	54	3.2	5.41×10^−14^
GO:0016709	Oxidoreductase activity, acting on paired donors, with incorporation or reduction of molecular oxygen, NAD or NADPH as one donor, and incorporation of one atom of oxygen	17	3.0	1.99×10^−5^

### Homologues of somatic embryogenesis-related genes are differentially methylated between NEC and EC

**Fig. 5.**
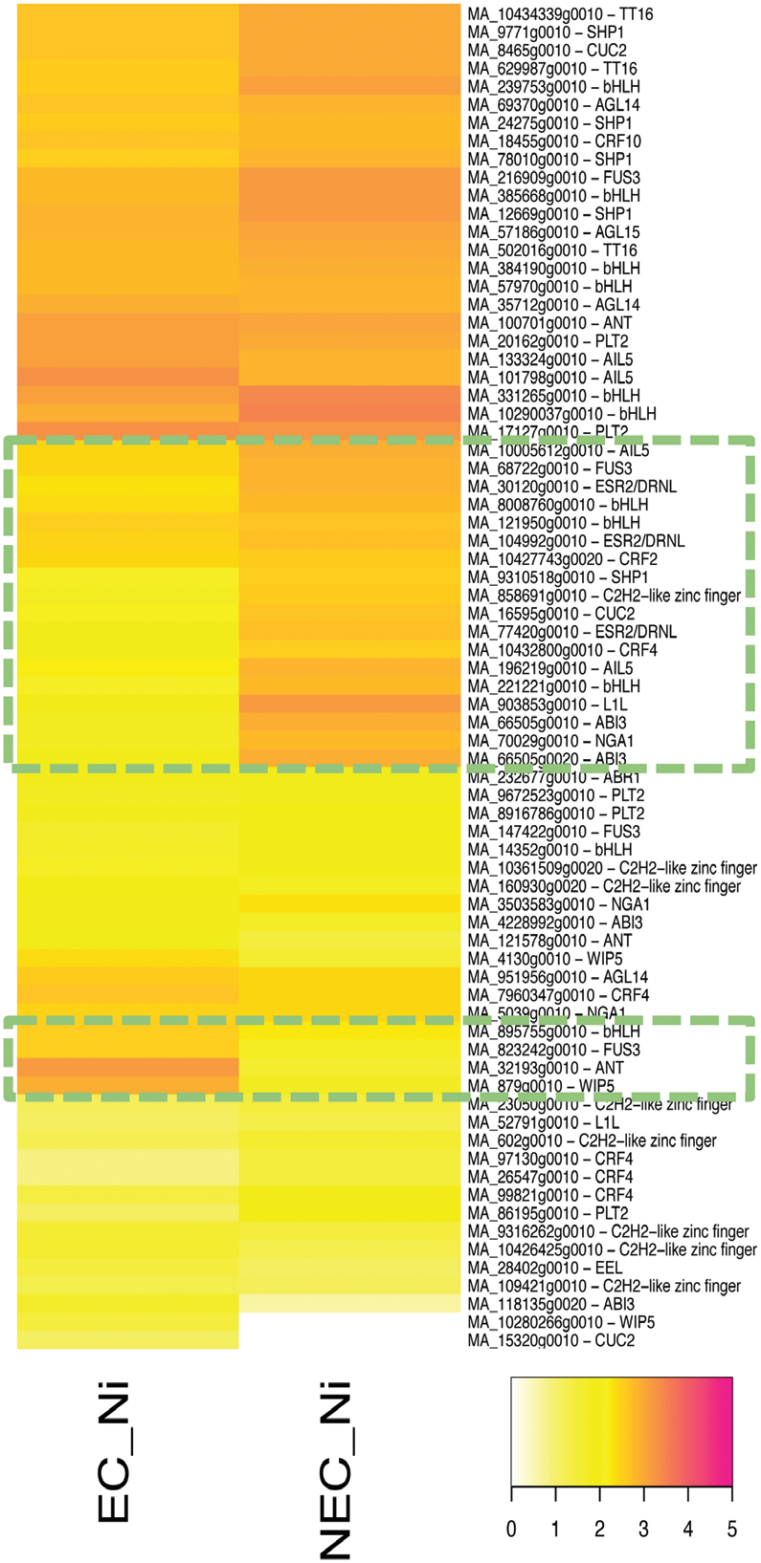
Differential accumulation of H3K27me3 on homologues of genes related to somatic embryogenesis. Heatmap shows the H3K27me3 level of homologous genes of transcription factors highly expressed in Arabidopsis somatic embryogenesis.

Studies in angiosperm species have identified several key regulators of somatic embryogenesis, among which are *PLETHORA 4/BABY BOOM* (*PLT4/BBM*) and *PLETHORA 5/EMBRYO MAKER* (*PLT5/EMK*), which are AP2-type transcription factors (Boutilier *et al.*, 2002; Tsuwamoto *et al.*, 2010). In addition, *AGAMOUS-LIKE 15* (*AGL15*) and the *LEAFY COTYLEDON* genes *LEC1* and *LEC2* are also known embryonic markers, and overexpression of these genes increases the embryogenic potential of Arabidopsis vegetative tissues (Lotan *et al.*, 1998; Stone *et al.*, 2001; Harding *et al.*, 2003; Thakare *et al.*, 2008). Beside these genes, we also investigated homologues of other somatic embryogenesis-related transcription factors that were identified in a transcriptome analysis of Arabidopsis somatic embryogenesis (Wickramasuriya & Dunwell, 2015; Mozgová *et al.*, 2017). To explore key regulators of somatic embryogenesis competency in gymnosperms, we compared the levels of H3K27me3 in somatic embryogenesis-related gene homologues, between non-induced NEC and EC samples (Fig. 5). Nearly half of the genes showed high H3K27me3 levels in both non-induced NEC and EC, whereas, gene subsets containing homologues of *ABI3* (*MA_66505g0020)*, *DRNL* (*MA_30120g0010*, *MA_104992g0010*, *MA_77420g0010*), *CRF4* (*MA_10432800g0010*), *CUC2* (*MA_16595g0010*), and *FUS3* (*MA_68722g0010*) accumulated higher levels of this histone mark in NEC. Interestingly, a second homologue of *FUS3* (*MA_823242g0010*) and *WIP5* (*MA_849g0010*), as well as one of the *ANT* homologues (*MA_32193g0010*) had higher H3K27me3 levels in EC (Fig. 5). H3K27me3 modification is generally associated with gene repression. Consistently, expression of genes with high H3K27me3 in NEC was strongly repressed in NEC (see Supplementary Fig. S5). Likewise, genes with high H3K27me3 in EC were transcriptionally repressed in EC (Supplementary Fig. S5). Thus, many of the somatic embryogenesis-related gene homologues showed differential H3K27me3 enrichment between NEC and EC, possibly explaining the difference of somatic embryogenesis potential among these tissues.

## Discussion

### CUT&RUN as a suitable tool to profile large genomes

So far, most studies in chromatin profiling in plants have been limited to relatively small genomes (e.g. different angiosperms, a moss, and a liverwort) (Zhang *et al.*, 2007; Widiez *et al.*, 2014; Hussey *et al.*, 2017; Latrasse *et al.*, 2017; Chica *et al.*, 2017; Lü *et al.*, 2018; Montgomery *et al.*, 2020). However, genome-wide analysis in gymnosperms has remained a challenge due to the large genome sizes of these species (Pellicer *et al.*, 2018). Here, we successfully applied the CUT&RUN method to perform a genome-wide detection of H3K27me3 in Norway spruce. The lack of a complete genome assembly prevents us from providing a chromosomal map of H3K27me3 distribution; nevertheless, we identified genes marked by this modification and can show that the H3K27me3 modification is localized in genic regions of the Norway spruce genome, similar to what is observed in other plant species (Fig. 2). Our validation of the CUT&RUN method will facilitate an in-depth analysis of the distribution of H3K27me3 once a complete spruce genome assembly is available and open the door for epigenetic profiling studies in other gymnosperm species.

### Conservation of H3K27me3 targets in Norway spruce and angiosperms

We found that some H3K27me3 target genes are similar between Arabidopsis and Norway spruce, supporting the conservation of the epigenetic mechanisms regulating these genes. At the same time, gymnosperm-specific genes also show H3K27me3, suggesting that H3K27me3 is an important epigenetic player in the regulation of species-specific pathways (Fig. 3C). Although the *cis*-elements associated with H3K27me3 deposition in Norway spruce are not similar to those described in Arabidopsis, AT-rich motifs and REF6 DNA-binding motifs were detected in our dataset. This co-localization of REF6 binding motif-like sequences and H3K27me3 suggests that, similarly to Arabidopsis, the H3K27me3 catalysis mechanism could be based on *cis*-regulatory sequences. Nevertheless, this remains to be formally tested: considering that the H3K27me3 distribution is usually broader than the PRC2 binding site itself (Xiao *et al.*, 2017), the H3K27me3 signal peak might not be a good predictor for *cis*-regulatory elements for H3K27me3 target genes. Therefore, a high-quality genome assembly and direct binding assays using PRC2 components will provide a better resolution for the identification of *cis*-regulatory elements such as the PREs identified in Arabidopsis. A previous study of chromatin conformation in a relatively large genome revealed that H3K27me3 is associated with chromatin domain borders (Dong *et al.*, 2017). Further analysis by Hi-C could provide a more complete view of H3K27me3 function in this large genome.

### H3K27me3 target genes underlie differences in somatic embryogenesis potential

Previous empirical evidence showed that non-induced EC and NEC have different embryogenic potentials. This study validates these observations at the molecular level and adds a possible functional explanation. Here we detected that non-induced EC and NEC had largely different genes being marked by H3K27me3 (Fig. 4), and that non-induced NEC has a H3K27me3 profile that more closely resembles a seedling hypocotyl, indicating that the H3K27me3 profiles might be inherited from original explants to some extent. Although we could not determine the H3K27me3 profile of immature zygotic embryos due to limited source material, we speculate that, as in NEC, the low H3K27me3 observed in EC could be due to an already low accumulation of H3K27me3 in immature zygotic embryos. The megaspore mother cell of Arabidopsis has lower H3K27me3 than the surrounding sporophytic cells, suggesting that there is a reduction of H3K27me3 during female gametogenesis (She *et al.*, 2013). Additionally, in Arabidopsis, it has been shown that PRC2 depletion followed by 2,4-D exposure is required for efficient somatic embryogenesis (Mozgová *et al.*, 2017). Our observations that EC contains reduced levels of H3K27me3 and that a preculturing in 2,4-D-containing medium is essential for somatic embryogenesis in Norway spruce show that this developmental process might be similarly controlled in angiosperms and gymnosperms. Furthermore, it points to 2,4-D as an important component that has the ability to promote chromatin reprogramming in an already predisposed tissue, such as EC, allowing for the somatic embryogenesis program to be completed.

In some angiosperm species, it has been reported that heterochromatic epigenetic marks are associated with SE initiation rate. For example, perturbations of DNA methylation or H3K9me2 by chemicals facilitate embryogenesis from microspores in *Brassica napus* and *Hordeum vulgare* (Solís *et al.*, 2015; Berenguer *et al.*, 2017). A recent study reported that DNA methylation on the target sequences of REF6, which is a H3K27me3 demethylase, decreases REF6 binding (Qiu *et al.*, 2019). If the global reduction of H3K27me3 in EC is associated with histone demethylase activity, it would be interesting to investigate whether loss of DNA methylation might be required preceding H3K27me3 reduction.

Associations between peroxidase activity and embryogenic potential have been observed in both angiosperms and gymnosperms (Kochba *et al.*, 1977; Hrubcová *et al.*, 1994; Kormutak & Vookova, 2001; Neves *et al.*, 2003; Gallego *et al.*, 2014). It is therefore interesting that GO terms related to (per)oxidase activities were enriched in response to somatic embryo induction (Table 3). Also genes involved in cell wall modification were significantly enriched among genes responding to somatic embryo induction (Table 3). Cell wall modification and the resulting cell wall dynamics are likely preceding organ initiation (Peaucelle *et al.*, 2011; Francoz *et al.*, 2015). Thus, the observed changes in H3K27me3 foreshadow the developmental processes of somatic embryo induction.

Generally, somatic embryogenesis takes 6–8 weeks after somatic embryo induction. We showed that both EC and NEC altered their H3K27me3 profiles only 1 week after somatic embryo induction, and that NEC and EC showed a different H3K27me3 accumulation profile in response to somatic embryo induction. While the H3K27me3 profile of induced EC resembled that of non-induced EC, NEC showed drastic changes in H3K27me3 target genes after induction. Considering that NEC reduces its proliferation and turns brown 2–3 weeks after SE induction, these large changes in the H3K27me3 profile might reflect a senescence program of the cells (Fig. 1). Indeed, in Arabidopsis, H3K27me3 is also associated with senescence-regulated genes (Ay *et al.*, 2009; Brusslan *et al.*, 2012).

In this study, we demonstrated that the genome-wide level of H3K27me3 in EC is extensively lower than in NEC and seedlings. Low H3K27me3 levels, together with the action of phytohormones such as 2,4-D might be the essential qualifications ensuring somatic embryogenesis potential in gymnosperms as well as in angiosperms (Mozgová *et al.*, 2017). Validation of this hypothesis, coupled with further studies of the genes affected by H3K27me3 during somatic embryogenesis, could open the possibility of manipulating this developmental process, increasing the efficiency of somatic embryogenesis protocols, or even improving the competency of recalcitrant tissues.

## Supplementary data

The following supplementary data are available at *JXB* online.

Fig. S1. Morphology of EC and NEC 2 weeks after somatic embryo induction.

Fig. S2. Percentage of genes with H3K27me3 modiﬁcation in each auxin-related gene family.

Fig. S3. Phylogenetic analysis of putative H3K27me3 methyltransferases in gymnosperms.

Fig. S4. Identification of *cis*-element sequences associated with H3K27me3 peaks.

Fig. S5. Expression of representative SE-related genes is anti-correlated with their H3K27me3 level.

Table S1. Sequencing data list.

Table S2. List of primers used in this study.

eraa365_suppl_Supplementary_Material

## Data Availability

All raw and processed sequencing data generated in this study have been submitted to the NCBI Gene Expression Omnibus (GEO; https://www.ncbi.nlm.nih.gov/geo/) under accession number GSE145331. Mapping data of each tissue are listed in Supplementary Table S1.
