# Supplementary material for: Polycomb Repressive Complex 2-mediated histone modification H3K27me3 is associated with embryogenic potential in Norway spruce
Source: J Exp Bot. 2020 Sep 7;71(20):6366–78. doi: 10.1093/jxb/eraa365 (PMC7586741; doi:10.1093/jxb/eraa365)
Supplement: eraa365_suppl_Supplementary_Material [file eraa365_suppl_supplementary_material.pdf]

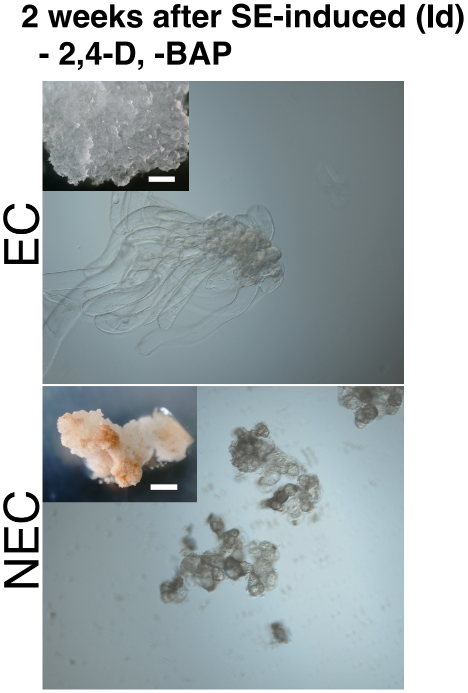

Fig. S1 Morphological observations of EC and NEC 2 weeks after somatic embyogenesis induction.

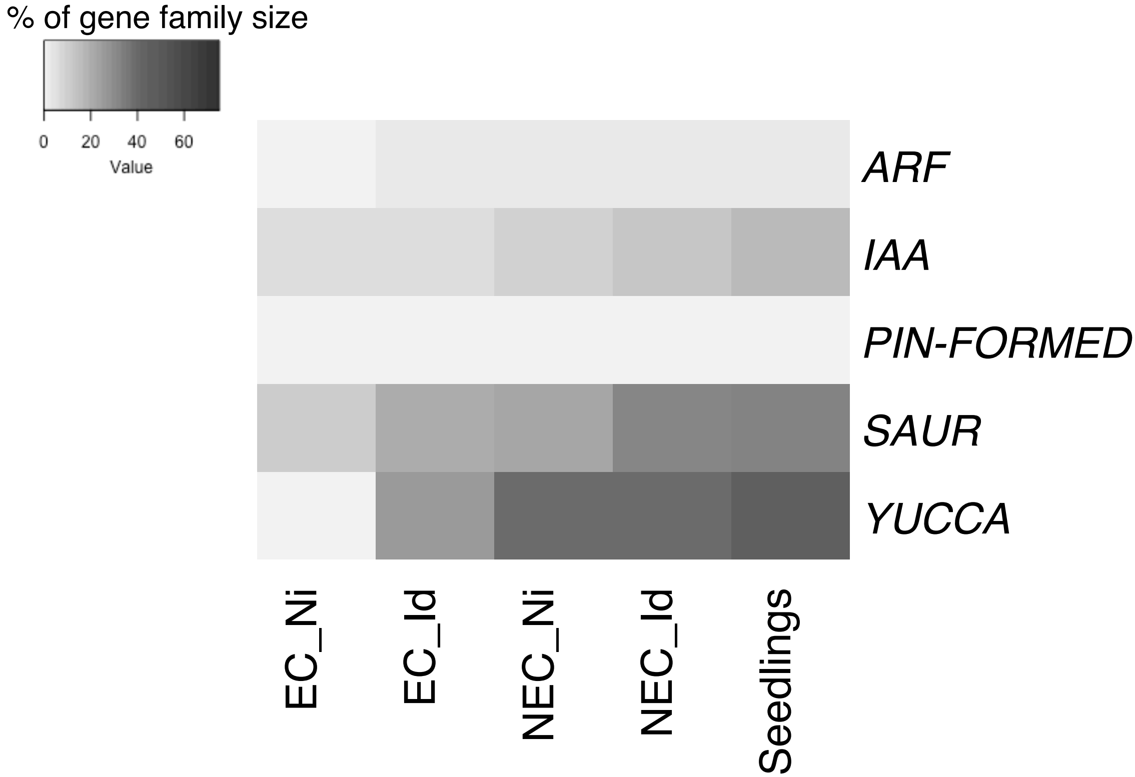

Fig. S2 Percentage of genes with H3K27me3 modification in each auxin-related gene family

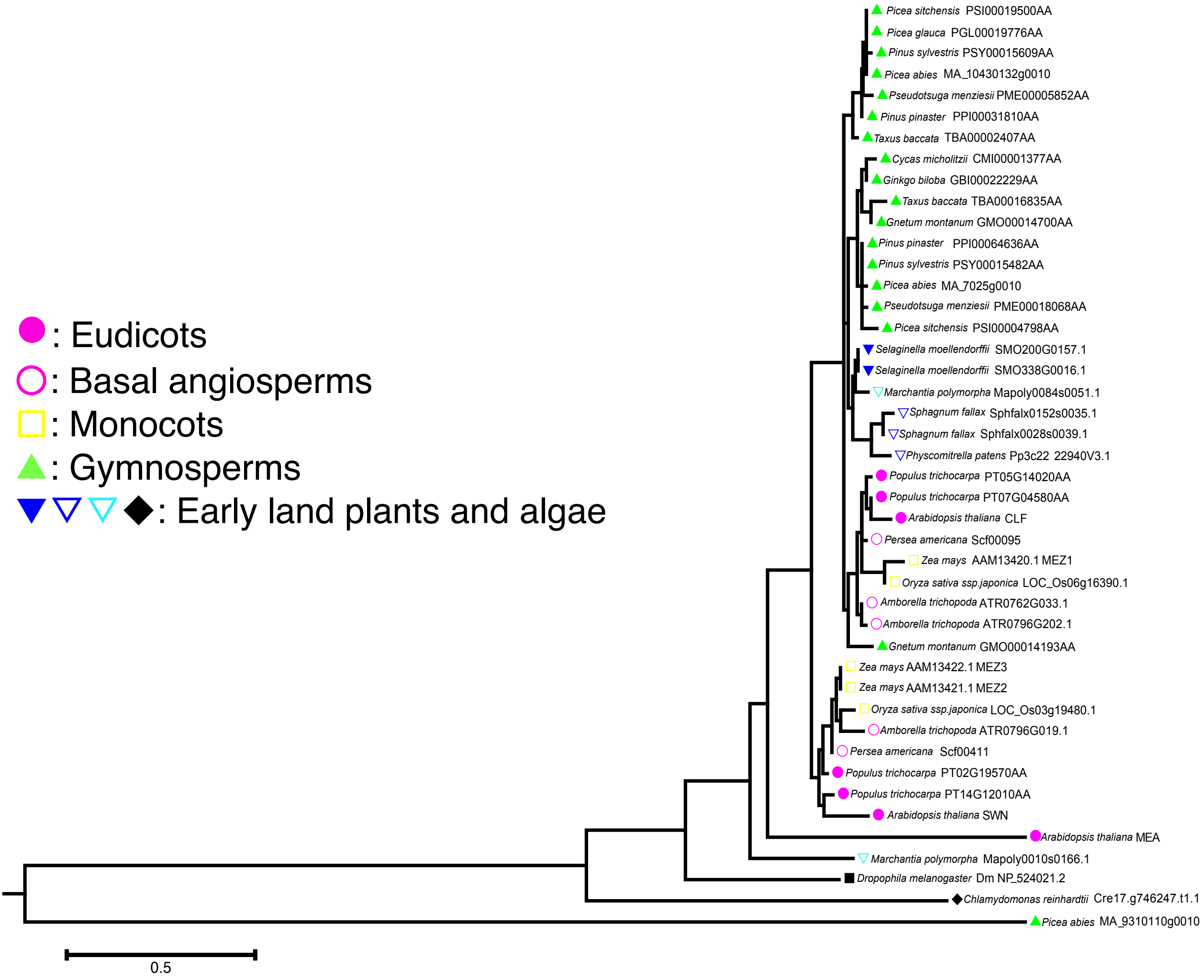

Fig. S3 Phylogenetic analysis of putative H3K27me3 methyltransferases in gymnosperms

Motif sequences at H3K27me3 peaks in SEC_Ni

MOTIF_INDEX	MOTIF_SOURCE	MOTIF_ID	E-VALUE	MOST_SIMILAR_MOTIF
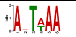	DREME	ANTWAA	2.2e−475	MA0990.1 (EDT1)
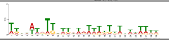	MEME	TTWWWTDTTDWTWWRWWTDTDTDWWWTTD	6.6e−421	MA1267.1 (AT5G66940)
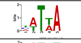	DREME	HWTWA	4.50E−240	
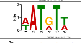	DREME	WATRTW	1.70E−68	
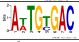	DREME	AWTGYGAC	1.40E−63	
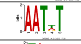	DREME	AATDT	6.30E−35	MA1162.1 (TCX2)
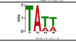	DREME	TAWW	9.20E−26	MA1383.1 (KAN2)
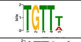	DREME	TGTTW	7.30E−12	MA1415.1 (REF6)
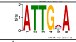	DREME	ATTGHA	1.50E−04	MA0008.2 (HAT5)
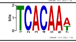	DREME	TCACAAW	8.10E−04	
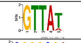	DREME	GTTAW	1.40E−03	MA1174.1 (MYB56)
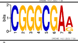	DREME	CGGGCGAR	7.70E−03	
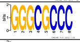	DREME	GGGCGCCC	1.40E−02	MA1410.1 (StBRC1)
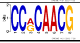	DREME	CCRCAACG	3.50E−02	

Motif sequences at H3K27me3 peaks in NEC_Ni

MOTIF_INDEX	MOTIF_SOURCE	MOTIF_ID	E-VALUE	MOST_SIMILAR_MOTIF
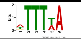	DREME	DTTTWA	9.3e−560	MA1161.1 (TSO1)
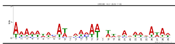	MEME	AAAWWWAMAWHAAMAWDWWWAAWWHAWAV	7.9e−494	MA1267.1 (AT5G66940)
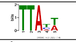	DREME	TTAHW	8.1e−319	
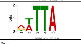	DREME	DWTTA	7.80E−96	MA1353.1 (AT1G72740)
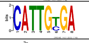	DREME	CATTGYGA	2.20E−48	
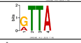	DREME	RTTA	2.10E−44	MA1293.1 (MYB57)
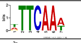	DREME	DTTCAAW	2.50E−43	
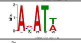	DREME	AHATW	5.40E−31	
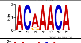	DREME	ACRAACA	2.30E−12	
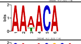	DREME	AAWACA	9.20E−07	MA1415.1 (REF6)
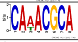	DREME	CAWACGCA	1.90E−04	
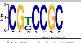	DREME	CGYCCGC	2.50E−04	
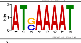	DREME	ATSAAAAT	1.70E−03	
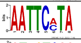	DREME	AATTCMTA	1.10E−02	
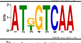	DREME	ATKGTCAA	2.10E−02	MA1077.1 (WRKY18)
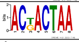	DREME	ACKACTAA	2.20E−02	
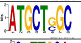	DREME	ATGCTSGC	3.80E−02	MA1345.1 (bZIP48)
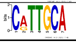	DREME	CRTTGCA	3.90E−02	

Fig. S4 Identification of *cis*-element sequences associated with H3K27me3 peaks

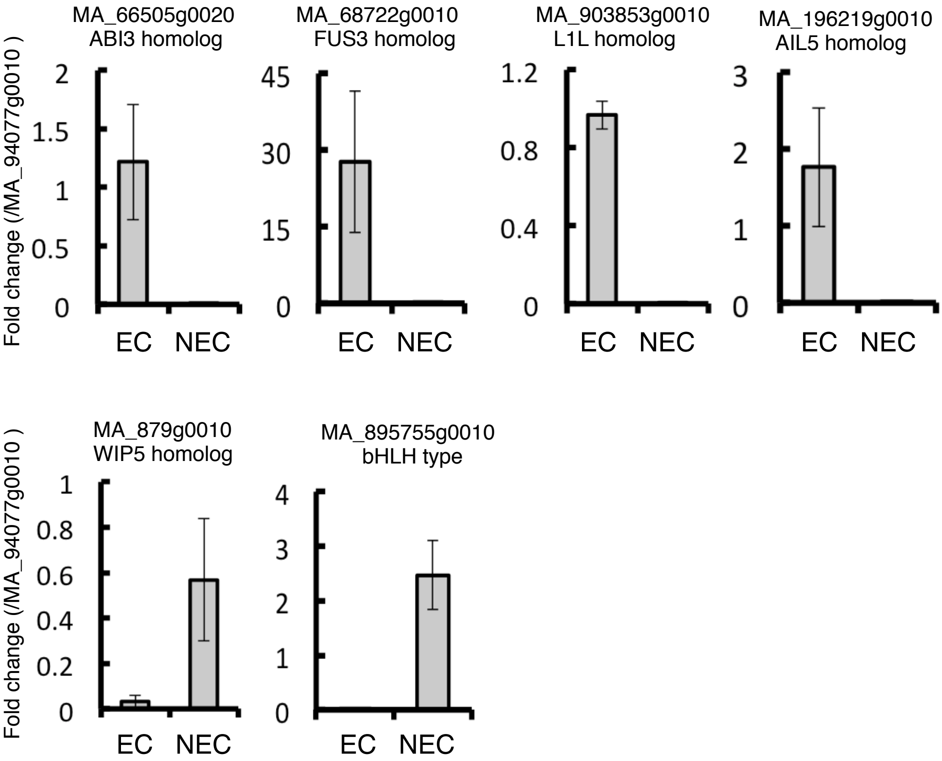

Fig. S5 Expression of representative SE-related genes is anti-correlated with their H3K27me3 level.

Expression is shown relative to a reference gene (MA_94077g0010). Shown are means ± standard deviation from 3 biological replicates before somatic embryo-induction.

Table S1. Sequencing data list

Sequence data ID	Sample name	No. of Raw reads	% of mapped reads	No. of mapped read	No. of distinct reads	Antibody
GSM4314896	Seedlings	89,145,873	59.6	53,163,364	50,026,807	K27me3
GSM4314897	EC_Ni	86,892,097	59.1	51,316,007	46,924,069	K27me3
GSM4314899	EC_Id	100,319,350	60.9	61,083,280	55,054,042	K27me3
GSM4314900	NEC_Ni	79,559,707	58.9	46,860,417	44,294,651	K27me3
GSM4314902	NEC_Id	95,399,520	61.1	58,319,692	53,983,570	K27me3

Table S2. List of primers used in this study.

Experiments	Name	Sequence (5′– 3′)
RT-qPCR		
	MA_94077g0010__RTqPCR_F1	CCAAGATCCAGGACAAGGAA
	MA_94077g0010__RTqPCR_R1	TTCCGGTAAGGGTTTTGACA
	MA_196219g0010_F1	TTATCTGGGAACATTCAGTTCAGA
	MA_196219g0010_R1	GGGCAATATGCAGTTTTCAATAGT
	MA_68722g0010_F1	GTACGTGGAGATTTCATCATGGTA
	MA_68722g0010_R1	ACTGAAAGCTGATAGGGAGACTTG
	MA_879g0010_F1	CAATCGCTATAACAACATGCAGAT
	MA_879g0010_R1	GTACAGCAGTAGCATGGAAGTCTC
	MA_895755g0010_F1	TGATAGGGTCTTTTACAGCATTCA
	MA_895755g0010_R1	ATGGAAGTTTTCCCTTCATCAAT
	MA_66505g0020_F1	GAGGACATTGTAACGTCTCGAGTA
	MA_66505g0020_R1	CGCCAGTATTTTCAAGCAGATACA
	MA_903853g0010_F1	ACAGAGAAATCGAAGGTGATCACA
	MA_903853g0010_R1	CAGCAGACAAATTAGCTAGAGCAC
